# Editor's Letter-Box

**Published:** 1897-05-22

**Authors:** 


### Editor's Letter-Box.

[Our Correspondents are reminded that prolixity is a great bar to publication, and that brevity of style and conciseness of statement greatly facilitate early insertion.]

Mr. Alfred A. Millward, Clerk to Guardians of the parish of St. Pancras, writes: I am desired by the Guardians of this parish to request that you will kindly afford space in your extensively-circulated paper to correct a misapprehension conveyed in the article headed "Nursing in Workhouses," which appeared in the issue of the 8th instant, so far as it relates to St. Pancras Infirmary. It is evident that the return made to the House of Commons, upon which the article was based, was not prepared in a uniform manner in all the Metropolitan parishes and unions. The particulars from this parish were given on the assumption that no nurses should be included under the heading of trained who had not been trained elsewhere before entering the service of the Guardians; and, as a result of this misunderstanding, we excluded those nurses who had been, or were still being, trained at our own infirmary. Of the 50 probationers employed at St. Pancras Infirmary, certified for 542 patients, 34 are in their second or third year; and might, as is now apparent, have been returned as "nurses," that course having been adopted elsewhere. I believe that in St. Marylebone Infirmary probationers are promoted to be "staff nurses" at the end of their first year. At the St. Pancras Infirmary they remain under the title of "probationer" for three years. For a fair comparison our return, therefore, should probably have been made out as follows: Night superintendents, 2; head nurses, 7; staff nurses, 34; probationers, first year, 16. In regard to the nature of the training which, in our institution is required before the title of "nurse" is granted. I should state that the probationers have to undergo successful examinations in nursing by an examiner specially appointed from outside, for which they have to prepare themselves by attending the periodical lectures and demonstrations given by the medical officers and the matrons. I enclose extracts from the most recent reports of the independent examiner, Dr. G. B. Thower White, of 112, Harley Street, W., showing the satisfactory results obtained by this system. I also enclose printed statements giving the numbers and duties of the nursing staff of the infirmary in force since 1894-5, from which it will be seen that the Guardians of St. Pancras have made great progress towards providing the sick poor under their charge with the ideal system of nursing so ably and persistently advocated through your valuable journal. This letter would be incomplete without an allusion to the fact that the St. Pancras Infirmary now possesses its "Nurses' Home," which was erected in 1893, with the most modern arrangements for the comfort of the nursing staff, at a cost of about £7,000.

*** Our figures were taken, as we stated, from the parliamentary return. We are very glad to find that the condition of affairs at the St. Pancras Infirmary is in every way so much better than was there stated.—Ed. *T.H.*

